# Epidemiology of Lyme disease, a growing tick-borne disease of concern, in Japan from May 2013 to March 2024: a descriptive study

**DOI:** 10.1016/j.ijregi.2026.100919

**Published:** 2026-05-21

**Authors:** Kohei Goto, Hirofumi Kato, Minako Kanesaki, Natsuko Nakamura, Ayano Orime, Chiaki Ikenoue, Kozue Sato, Tomoe Shimada, Yu Takizawa, Takuri Takahashi, Taro Kamigaki, Hiroki Kawabata, Tomimasa Sunagawa

**Affiliations:** 1 Field Epidemiology Training Program, National Institute of Infectious Diseases, Japan Institute for Health Security, Tokyo, Japan; 2 Department of Pediatrics, Graduate School of Medicine, Gifu University, Gifu, Japan; 3 Center for Public Health Action in Applied Epidemiology, National Institute of Infectious Diseases, Japan Institute for Health Security, Tokyo, Japan; 4 Department of Bacteriology-I, National Institute of Infectious Diseases, Japan Institute for Health Security, Tokyo, Japan; 5 Department of Infectious Disease Surveillance, National Institute of Infectious Diseases, Japan Institute for Health Security, Tokyo, Japan

**Keywords:** Lyme disease, Epidemiology, Japan, Surveillance, Tick-borne disease

## Abstract

**Objectives:**

Lyme disease (LD) is a globally distributed zoonosis; however, limited epidemiological data are available from Japan. This study aimed to elucidate the characteristics of LD cases reported through the Japanese national surveillance system.

**Methods:**

We conducted a retrospective descriptive study based on data from the National Epidemiological Surveillance of Infectious Diseases for the fiscal years 2006-2023.

**Results:**

Overall, 265 LD cases were reported, ranging from five in 2008 to 28 in 2023 (median: 12.5), including 216 domestic, predominantly from Hokkaido, and 43 imported cases. The median annual notification rate for domestic cases was 0.94 per 10 million population (range: 0.39-2.14). The median age was 53 years, and 62% were male. No fatal cases were reported. Most infections occurred between May and September. Common clinical features of the cases included erythema migrans, fever, and neurological symptoms. The median duration from onset to diagnosis was 28.5 days (interquartile range: 16-45 days).

**Conclusions:**

The number of LD cases in Japan has increased, with cases predominantly occurring in middle-aged men from Hokkaido and other areas. Furthermore, patients having LD presented with non-specific symptoms, including neurological involvement, resulting in delayed diagnoses. These findings highlight the importance of increasing awareness of LD in Japan.

## Highlights

• An overview of the epidemiological and clinical characteristics of Lyme disease in Japan.
• Gradual increase in the number of reported cases in Japan.
• At-risk populations including pediatric patients and middle-aged men, with delayed diagnosis.
• Importance of increasing awareness among healthcare personnel, residents, and visitors of affected areas.

## Introduction

Lyme disease (LD), also known as Lyme borreliosis, is an emerging tick-borne disease caused by *Borrelia burgdorferi* sensu lato, which is transmitted to humans by bite of *Ixodes* tick [1]. LD is also known as zoonosis, and its reservoir hosts include rodents, birds, and other vertebrates [1]. Since LD was first reported by a Yale research group in 1977 [2], its cases have been reported in the Northern Hemisphere, mainly in North America and Europe. Tens of thousands to hundreds of thousands of LD cases are still reported each year in these areas, and the number of reported cases is increasing annually over time [3,4]. In Europe, the major pathogens are *B. garinii, B. afzelii*, and *B. bavariensis*, in addition to *B. burgdorferi*, and the primary tick vector is *Ixodes ricinus*. In North America, the main pathogen is *B. burgdorferi*, and the primary vectors are black-legged ticks, such as *I. scapularis* and *I. pacificus*. In addition, antibodies against LD have been detected in human serum in Asian countries; the seroprevalence rates were 12.1% and 5.7%, respectively, by enzyme immunoassay or indirect fluorescent antibody test and two-tier testing [5]. Many LD cases are believed to be distributed worldwide, including Asia.

Most patients having LD present with mild symptoms; however, if left untreated, the disease can be severe, and in rare cases, fatal [6]. The clinical course of LD is divided into three stages: early localized, early disseminated, and late. In the early localized stage, the most common clinical manifestation is erythema migrans (EM), characterized by a gradually expanding erythematous skin rash around the tick bite site. It is sometimes accompanied by flu-like symptoms, such as muscle pain, joint pain, headache, fever, chills, and fatigue. In the early disseminated stage, owing to the dissemination of bacteria from the tick bite site, various symptoms are observed, including skin manifestations, neurological symptoms, cardiac disease, and arthritis. In the late stage, the infection can disseminate via the bloodstream to parts of the body when untreated or inadequately treated with antibiotics, leading to serious manifestations affecting the nervous system, joints, or skin (e.g. acrodermatitis chronica atrophicans), months to years after a tick bite.

The avoidance of tick bites and post-exposure prophylaxis are essential measures to prevent LD infection. Vaccination has also been considered as a preventive strategy. In 2002, a human vaccine (LYMErix) was approved for use against LD in the USA [1]. However, owing to concerns about side effects and low demand, the manufacturer voluntarily discontinued sales [6]. Although several vaccines are currently under development, none have been approved [6].

The first case of LD in Japan was reported in 1986 [7]. The main causative pathogen in the country is *B. bavariensis*, and the primary vector is *I. persulcatus* [8], which is mainly distributed in Russia and East Asia. Recently, there has been considerable public health interest in tick-borne diseases in Japan because tick-borne diseases, such as severe fever with thrombocytopenia syndrome (SFTS) and Japanese spotted fever (JSF), have been increasing or spreading throughout the country [9]. Although LD has been a notifiable disease in Japan since 1999, comprehensive situational analysis has been limited [10]. This study aimed to elucidate the characteristics of LD cases reported in Japan based on time, place, and person, using national surveillance data. This study provided a recent epidemiological analysis of LD in Japan.

## Methods

### Surveillance of LD in Japan

In Japan, the National Epidemiological Surveillance of Infectious Diseases (NESID) for LD was initiated in 1999 under the Act on the Prevention of Infectious Diseases and Medical Care for Patients with Infectious Diseases, which is commonly referred to as the Infectious Disease Control Law. LD is classified as a category IV notifiable infectious disease in the country, and laboratory-confirmed symptomatic and asymptomatic LD cases must be promptly reported upon diagnosis by a physician. The estimated place of infection was determined based on the location where the physicians estimated that the infection had occurred, considering case information, including medical and social histories. In the NESID system, symptoms and outcomes at the time of notification have been reported; therefore, the final symptoms and outcomes remain unknown unless voluntarily reported subsequently, because a follow-up investigation is not mandated by the system. The case definition includes individuals confirmed by one of the following laboratory methods: (1) isolation of the pathogen or detection of bacterial deoxyribonucleic acid (DNA) sequences from skin lesions at the erythema site or from cerebrospinal fluid (CSF) in cases of meningitis or encephalitis, or (2) confirmation of elevated Lyme-specific antibodies in serum by immunoblotting.

### Testing method

In Japan, laboratory diagnostic tests for LD are conducted at a limited number of facilities, including several local public health institutes and the National Institute of Infectious Diseases, Japan Institute for Health Security (NIID-JIHS). Laboratory tests are performed in accordance with the standard operating protocol published by the NIID-JIHS, which follows the recommendations (conventional two-tiered testing) of the U.S. Centers for Disease Control and Prevention [11]. The cost of these tests is covered by public expenditure, but not by health insurance.

The most common diagnostic method used is the measurement of anti-*Borrelia* antibodies in the serum. Commercial test kits for supplemental immunoblot, such as RecomLine *Borrelia* assay (Mikrogen Diagnostik GmbH, Germany), are also used within the public health system. Polymerase chain reaction (PCR) detection of *Borrelia* DNA is performed using skin biopsy specimens, blood, or CSF in diagnostic laboratories. Because cases of co-infection with LD *Borrelia* and *B. miyamotoi*, which can cause relapsing fever borreliae, have been reported in Japan [12], diagnostic laboratories often use PCR methods capable of simultaneously detecting the genes for both LD and relapsing fever borreliae caused by *B. miyamotoi* [13].

Although the Barbour–Stoenner–Kelly medium can be used for culturing *Borrelia* [14], it is not widely used because the time to diagnosis is longer with this method than with PCR.

### Study design and data collection

Based on NESID data from May 2006 (fiscal year 2006; hereafter, FY2006) to March 2024 (FY2023), a retrospective and descriptive epidemiological study was conducted. The following information was extracted from the NESID database: demographics, occupation, travel history, estimated infection area, diagnostic methods, and clinical manifestations. The times from onset to the first visit and diagnosis were used when the dates of onset, first visit, and diagnosis were completely recorded, and the time series was consistent for evaluation. Notification rates were calculated using population census data from the Statistics Bureau of Japan, based on the population from October 1, 2005 to 2020 [15].

### Statistical analysis

The Mann-Whitney *U* test was used to compare times between LD cases with and without specific symptoms. The Mann-Kendall test was used for the assessment of significance of trends in annual LD cases. Statistical significance was set at *P* < 0.05. All analyses were conducted using STATA/SE 17.0. software (Stata Corporation, College Station, TX).

### Ethics

This study was exempt from an institutional ethics review as per the Japanese Infectious Disease Control Law, because it was conducted for public health purposes using national surveillance data.

## Results

**Figure 1:**
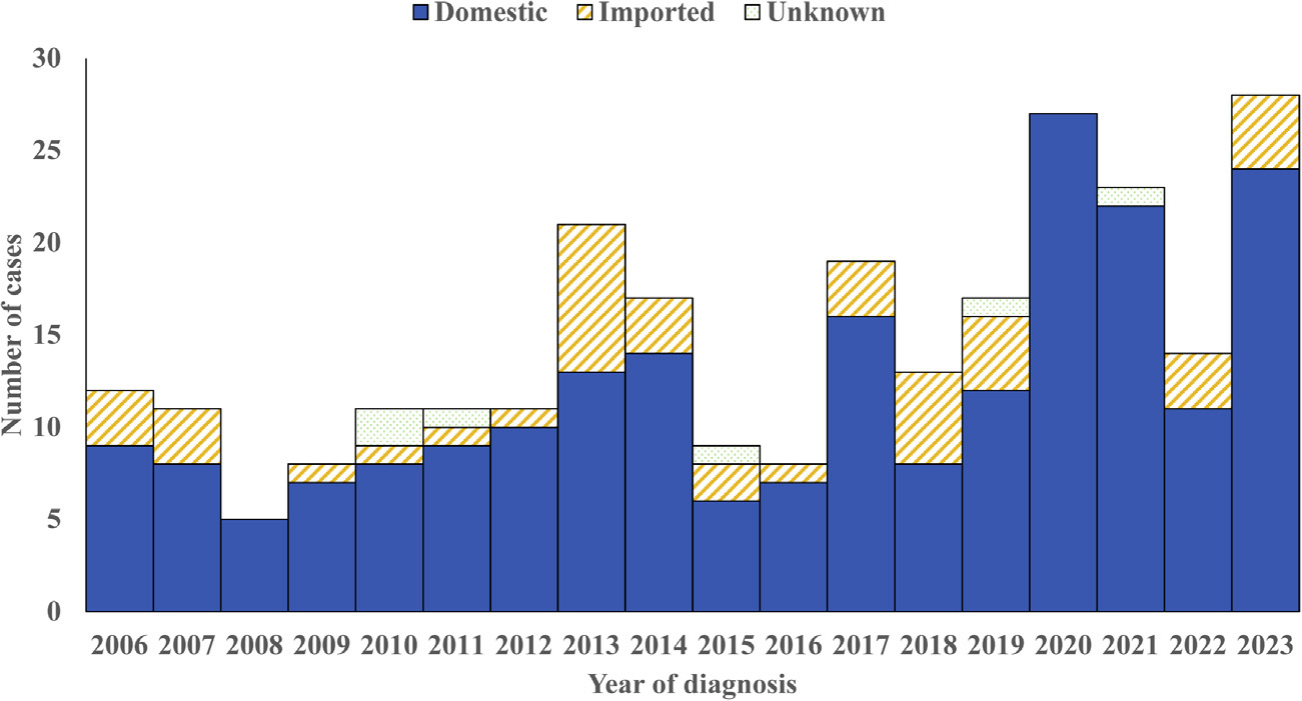
Epidemic curve of Lyme disease cases in Japan by year of diagnosis, based on the National Epidemiological Surveillance of Infectious Diseases, May 2006 (FY2006) to March 2024 (FY2023) (n = 265).Blue: domestic cases; yellow diagonal line: imported cases; and green dot: unknown cases. FY, fiscal year.

During the study period, 265 cases were reported using the NESID system. No fatal cases were reported at the time of notification. Of these, 216 (82%), 43 (16%), and six (3%) cases were domestic, imported, and of unknown origin, respectively (Figure 1). The median annual notification rate of domestic cases was 0.94 per 10 million population (range: 0.39-2.14).

Regarding the imported cases, the most commonly estimated areas of infection were the USA (16 cases, 37%), Germany (six cases, 14%), Sweden (four cases, 9%), and Poland (three cases, 7%). Of the reported domestic cases, the annual number increased slightly from nine in 2006 to 12 in 2019. However, since 2020, more than 20 cases have been reported (27, 22, and 24 cases in 2020, 2021, and 2023, respectively), with fluctuations. The Mann-Kendall trend analysis revealed a statistically significant increase in the annual number of domestic cases between 2006 and 2023 (Kendall’s tau = 0.47, *P* = 0.007), with a Sen’s slope of 0.81 cases per year (95% confidence interval: 0.23-1.30).

**Figure 2:**
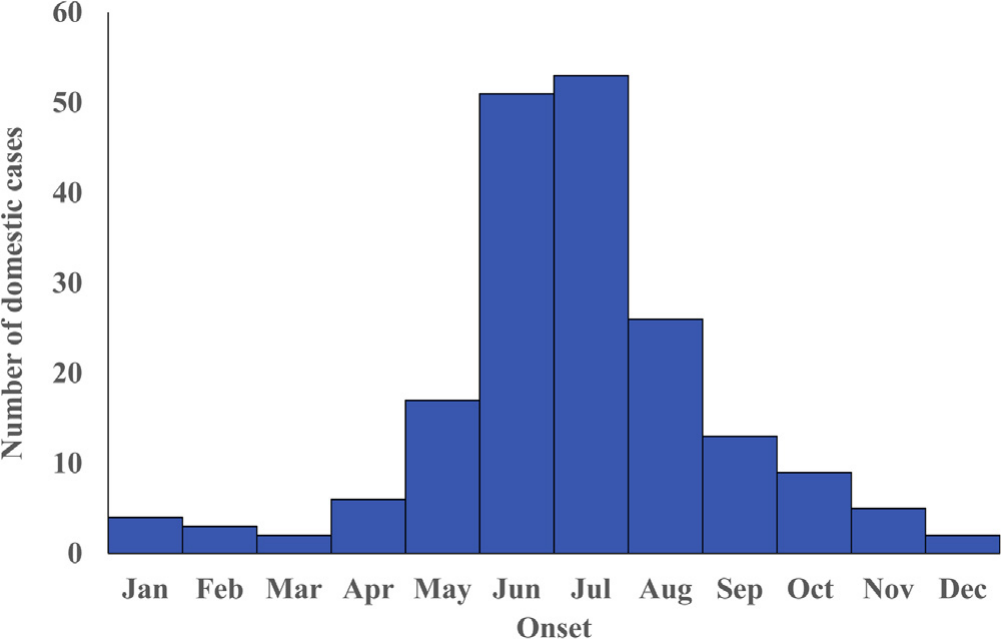
Epidemic curve of Lyme disease cases in Japan by onset month, based on the National Epidemiological Surveillance of Infectious Diseases, May 2006 (FY2006) to March 2024 (FY2023) (n = 191). FY, fiscal year.

**Figure 3:**
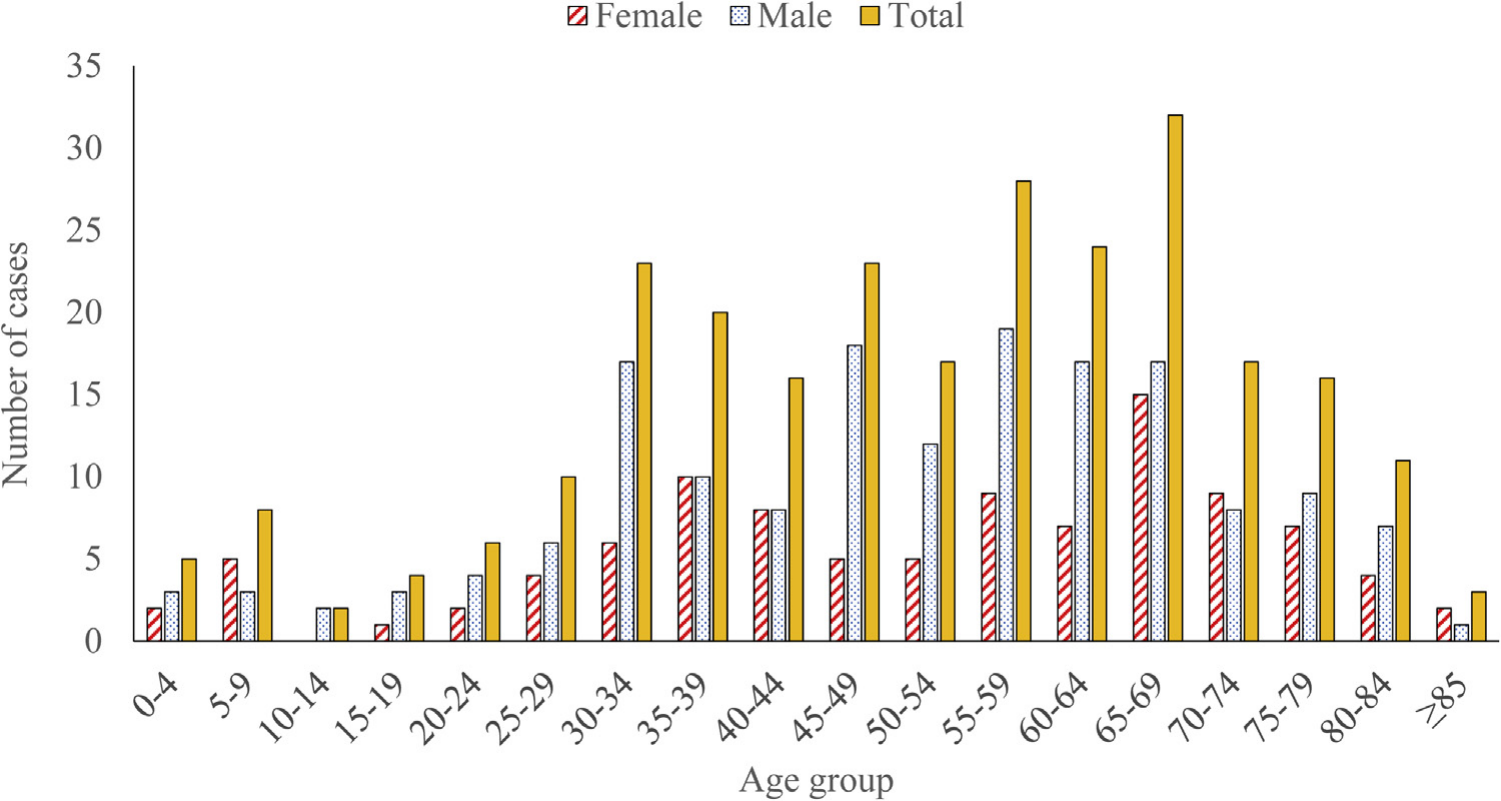
Age group and sex distribution of Lyme disease cases in Japan, based on the National Epidemiological Surveillance of Infectious Diseases, May 2006 (FY2006) to March 2024 (FY2023) (n = 265). FY, fiscal year.

Annually, most cases were reported from spring to autumn, with peaks in June and July (Figure 2). Of the reported cases, 164 (62%) were reported in male individuals, and the median age was 53 years (interquartile range [IQR]: 36-66 years). Cases were most commonly reported in individuals aged 60-69 years (103 cases, 39%), whereas those in individuals younger than 15 years (15 cases, 6%) were rare (Figure 3). Most cases were unemployed/retired workers (69 cases, 26%), followed by workers in agriculture, forestry, and fisheries (27 cases, 10%); public servants (17 cases, 6%), including 12 employed in the self-defense forces and one in the forestry agency; and students (14 cases, 5%). The median times from onset to the first visit, onset to diagnosis, and the first visit to diagnosis were 4 days (IQR: 1-10 days), 28.5 days (IQR: 16-45 days), and 20 days (IQR: 9-35 days), respectively. Furthermore, the median time from the first visit to diagnosis was 28 days (IQR: 15-44 days) for LD cases with EM and 41 days (IQR: 19-73 days) for those without EM, indicating a significant difference between the groups (*P* = 0.007).

**Figure 4:**
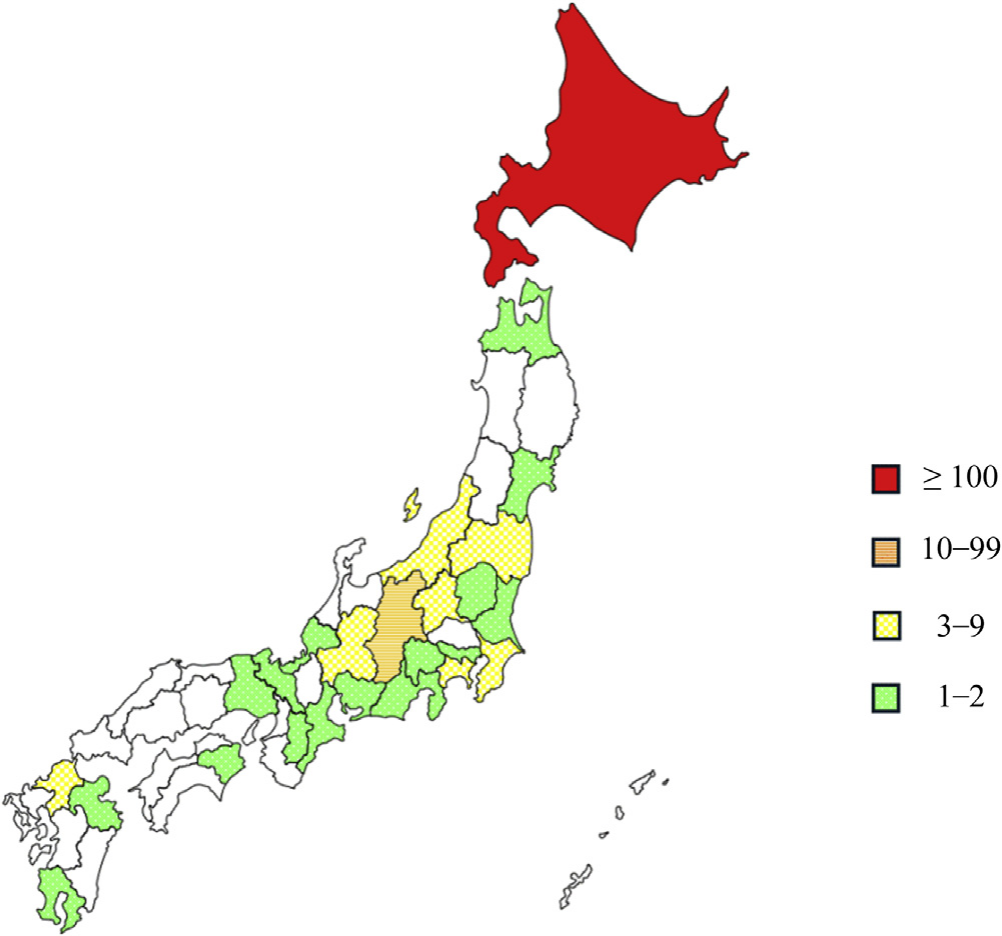
Geographic distribution of the estimated infection regions for Lyme disease cases in Japan, based on the National Epidemiological Surveillance of Infectious Diseases, May 2006 (FY2006) to March 2024 (FY2023) (n = 265). Red: ≥100 cases; orange: 10-99 cases; yellow: 3-9 cases; and green: 1-2 cases. FY, fiscal year.

The most common estimated places of infection were Hokkaido (161 cases, 75%), Nagano (11 cases, 5%), Niigata (five cases, 2%), and Gumma and Kanagawa (four cases, 2%) (Figure 4). Hokkaido accounted for approximately 50% of domestic cases in 2009; this percentage has increased over time, reaching approximately 90% by 2023.

Among the reported cases, 256 (97%) were symptomatic. The main symptoms observed in the early stage (stage I) were EM (178 cases, 70%), fever (115 cases, 45%), and myositis/myalgia (66 cases, 26%). Neurological symptoms and arthritis, which are often observed during the dissemination stage (stage II), were reported in 51 (20%) and 19 (7%) cases, respectively. Among the patients who presented with neurological symptoms, more detailed symptoms were designated in 25 cases. The most common neurological symptoms reported were peripheral nerve symptoms (14 cases), meningitis (11 cases), and facial nerve palsy (six cases). In the late stage (stage III), joint swelling specific to chronic arthritis and acrodermatitis chronica atrophicans were reported in eight cases (3%) and one case (<1%), respectively. The most frequently used diagnostic method was the serological test which detected elevated levels of anti-LD *Borrelia* antibodies (243 cases, 92%), followed by PCR (22 cases, 8%) and isolation (nine cases, 3%).

## Discussion

In this study, we described the detailed characteristics of LD cases in Japan by using national surveillance data. Similar to the increasing trends observed for other tick-borne diseases, such as SFTS and JSF, over time in Japan, the average number of domestic cases tended to increase, doubling from 2020 to 2023 compared to 2006-2009; most of the increased cases occurred in Hokkaido. The reason for this remains unclear, but one possible explanation is the increased awareness among healthcare personnel in Hokkaido. Relapsing fever caused by *B. miyamotoi*, which is transmitted by the same tick vector as LD, *I. persulcatus*, has been reported in Hokkaido since 2014 [12]. In addition, several tick-borne encephalitis cases have been reported since 2016 [16]. Furthermore, emerging tick-borne diseases such as SFTS and the Yezo virus disease have increasingly raised concerns in Japan, including Hokkaido [17]. These factors may have elevated the awareness of tick-borne diseases, especially in Hokkaido.

LD cases are reported mainly from spring to autumn, with peaks in June and July. Similar seasonal trends were found in countries in the Northern Hemisphere [3,4]. According to a study conducted in Hokkaido from 1980 to 2000, of 817 individuals who had tick bites, 499 (61%) were bitten in June and July [18], presumably because of an increase in outdoor occupational or recreational activities, such as farming, forestry, hunting, and hiking. Additionally, it has been reported that the number of collected *I. persulcatus* was higher in June and July in Hokkaido [19]. Collectively, both ticks and human activities may contribute to the seasonality of LD.

In this study, 216 domestic cases were reported across 25 prefectures (Figure 4). Of the domestic cases, 161 (75%) infections were estimated to have occurred in Hokkaido, followed by 11 (4%) in Nagano and 5 (2%) in Niigata. Though *I. persulcatus* is widely distributed in low and highlands in Hokkaido, it is also found in highlands at elevations of 1000-1500 m in other areas (Supplementary Figure 1). The distribution of human cases almost corresponded with that of ticks; however, the estimated place of infection partially differed from that of known tick habitats. This partial discrepancy may be underreported and should be investigated in future studies. Of the imported cases, most were estimated to have been acquired in the USA and Europe, where LD is endemic. Given the epidemiological situation in these regions and ongoing international travel, healthcare personnel should monitor the trends and distribution of LD both domestically and internationally.

In Japan, LD cases were characterized by a median age of 53 years and a male proportion of 62%. In contrast, the reported median ages were higher and male proportions were almost equal to female for other major tick-borne diseases: 75 years and 50% for SFTS [20], 71 years and 46% for JSF [21], and 69 years and 56% for Tsutsugamushi disease [22], respectively. These findings suggest that in Japan, LD tends to affect relatively younger individuals and more often male individuals, compared with other tick-borne infections. A systematic review and meta-analysis suggested that the incidence of LD was slightly higher in women than in men, without significant differences among Asian populations [5]. In contrast, it was reported that male individuals accounted for the majority of LD cases in the USA [23] and Canada [24]. Generally, male individuals tend to engage in more outdoor activities, use less protective clothing or repellents, and have fewer outdoor companion animals, leading to exposure to ticks [24]. Further investigation is needed to determine why the male-to-female ratio for LD differs from that for other tick-borne diseases and other countries, considering not only human behavior, but also differences in laboratory sensitivity and biological factors. Another notable difference from other tick-borne diseases is the age distribution of LD. Although unimodal trends have been observed for other tick-borne diseases, our study showed a bimodal pattern, with a small peak among pediatric LD cases and a larger peak among middle-aged LD cases. This finding was similar to that of a study conducted in the USA, which reported a bimodal age distribution among pediatric and older patients with LD [23]. Several Japanese studies have reported that tick bites are frequently observed among pediatric and older individuals in outpatient clinics, showing a bimodal age distribution, with large peaks in these age groups [18,25]. Increased medical attention to children owing to parental concerns may also have contributed to the observed pediatric peak, in addition to middle-aged individuals who may be more engaged in occupational or recreational activities in tick-infested environments [24].

The reported clinical characteristics were not substantially different from those reported previously [26]. EM was the most frequently reported clinical presentation, occurring in approximately 70% of cases. Flu-like illnesses were also reported, with 45% of the affected patients having fever and 26% having body aches. Neurological involvement is one of the most severe manifestations of LD. This study revealed that 20% of the reported cases involved neurological symptoms, such as central nerve disorder, peripheral neuropathy, and meningitis, which were observed during the early disseminated stage. A previous study suggested that Lyme neuroborreliosis might be underdetected in Japan [27]. Therefore, when encountering unexplained neurological symptoms, healthcare personnel in the affected areas should consider LD as a potential diagnosis. In this study, no fatal cases were reported, although it should be noted that fatal or nonfatal status at the time of notification was reported in the system without a follow-up investigation. Additionally, asymptomatic LD cases with tick bites accounted for approximately 3% of the reported cases. Given that a seroepidemiological study conducted in Asia and Japan reported a prevalence of approximately 5% [5], patients without typical symptoms but with a history of travel to affected areas and tick bites with *I. persulcatus* might be considered for LD testing.

In this study, the median times from onset to the first visit and diagnosis were 4 and 28.5 days, respectively. In contrast, the median time from first symptoms to treatment among LD cases was 6 days (range: 0-70 days) and 13 days (range: 0-13,890 days) in the USA [28,29] and 16.5 days in Europe [30], where diagnostic access and clinical awareness are generally higher than those in Japan. Furthermore, in Japan, the median time from onset to confirmed diagnosis of SFTS, a tick-borne disease, was 6 days (IQR: 5-8 days) [20]. Overall, despite early medical consultations for LD cases, diagnosis was delayed by approximately 3 weeks, likely owing to limited clinical awareness and a lack of widely used commercial tests, compared to those associated with other countries and diseases. In addition, our study showed that time to diagnosis was shorter in LD cases with EM as the specific symptom. A previous study also reported similar findings, indicating that LD could be diagnosed earlier in participants with EM [29]. To date, in Japan, serological tests for LD have been performed by several local public health institutes and the NIID-JIHS, making access to diagnostic testing difficult. To promote earlier diagnosis, it is essential to increase awareness of infection risks among both citizens and physicians, thereby encouraging earlier testing.

This study has some limitations. First, as described above, information, including symptoms and outcomes, was reported to the surveillance system at the time of notification; therefore, the final outcomes might not have been followed up, and symptoms occurring after notification were not captured by this system. Second, the estimated place of infection was determined based on physicians’ assessments, considering the medical and social histories of the cases. Third, similar to other reportable infectious diseases, LD is likely to be underestimated in surveillance systems. Limited laboratory access also contributes to the underreporting of LD. Despite these limitations, our study, based on nationally reported surveillance data, provides useful insights for identifying nationwide trends and characteristics of LD in Japan and can help guide public health interventions to reduce the risk of LD infection.

In conclusion, this study provided an overview of the epidemiological and clinical characteristics of LD in Japan, with seasonality. In recent years, the number of reported cases has gradually increased, and LD cases have been observed not only in Hokkaido but also in other regions. Furthermore, this study described a bimodal distribution of LD cases among pediatric individuals and middle-aged men, with delayed diagnosis. These findings highlight the importance of increasing awareness among healthcare personnel, residents, and visitors of affected areas, including those with few reported cases, regarding the current situation and the associated risk factors, thereby supporting more effective public health prevention efforts targeting these populations.

## Declaration of competing interest

The authors have no competing interests to declare.

## Appendix Supplementary materials

Supplementary Figure 1. Estimated geographic distribution of *Ixodes persulcatus* in Japan. This figure was created by MEDICAL FIG., a service of Medical Education, Inc., based on the reference (Nobuhiro Takata. Geographic distribution of ticks. Illustrated guide to pathogenic ticks. Kinpodo, INC; 1992. p. 113-135). Red hatched area, estimated habitat of *Ixodes persulcatus*; shade of green, difference in elevation; thick black line, region boundary; thin black line, prefecture boundary. Image, application 1

## References

[1] Smith RP. (2025). Lyme disease. Ann Intern Med.

[2] Steere A.C., Malawista S.E., Snydman D.R., Shope R.E., Andiman W.A., Ross M.R. (1977). An epidemic of oligoarticular arthritis in children and adults in three connecticut communities. Arthritis Rheum.

[3] Kugeler K.J., Schwartz A.M., Delorey M.J., Mead P.S., Hinckley AF. (2021). Estimating the frequency of Lyme disease diagnoses, United States, 2010-2018. Emerg Infect Dis.

[4] Sykes R.A., Makiello P. (2017). An estimate of Lyme borreliosis incidence in Western Europe†. J Public Health (Oxf).

[5] Song J., Dong Y., Zhang Y., Zhou G., Wu X., Gao L. (2025). Seroprevalence of Lyme disease in Asian human populations: a systematic review and meta-analysis. Vector Borne Zoonotic Dis.

[6] Bostic T.D., Hook S.A., Marx GE. (2025). Lyme disease vaccine acceptability among healthcare providers - United States, 2018 and 2022. Vaccine.

[7] Kawabata M., Baba S., Iguchi K., Yamaguti N., Russell H. (1987). Lyme disease in Japan and its possible incriminated tick vector, *Ixodes persulcatus*. J Infect Dis.

[8] Takano A., Nakao M., Masuzawa T., Takada N., Yano Y., Ishiguro F. (2011). Multilocus sequence typing implicates rodents as the main reservoir host of human-pathogenic *Borrelia garinii* in Japan. J Clin Microbiol.

[9] Yamaji K., Aonuma H., Kanuka H. (2018). Distribution of tick-borne diseases in Japan: past patterns and implications for the future. J Infect Chemother.

[10] Lee W.C., Lee M.J., Choi K.H., Chung H.S., Choe NH. (2019). A comparative study of the trends in epidemiological aspects of Lyme disease infections in Korea and Japan, 2011-2016. J Vector Borne Dis.

[11] Centers for Disease Control and Prevention (CDC) (1995). Recommendations for test performance and interpretation from the Second National Conference on Serologic Diagnosis of Lyme Disease. MMWR Morb Mortal Wkly Rep.

[12] Sato K., Takano A., Konnai S., Nakao M., Ito T., Koyama K. (2014). Human infections with *Borrelia miyamotoi*. Japan. Emerg Infect Dis.

[13] Barbour A.G., Bunikis J., Travinsky B., Hoen A.G., Diuk-Wasser M.A., Fish D. (2009). Niche partitioning of *Borrelia burgdorferi* and *Borrelia miyamotoi* in the same tick vector and mammalian reservoir species. Am J Trop Med Hyg.

[14] Takano A., Toyomane K., Konnai S., Ohashi K., Nakao M., Ito T. (2014). Tick surveillance for relapsing fever spirochete *Borrelia miyamotoi* in Hokkaido. Japan. PLoS One.

[15] Portal Site of Official Statistics of Japan (2025). Population census. https://www.e-stat.go.jp/en/stat-search?page=1&toukei=00200521.

[16] Yoshii K., Takahashi-Iwata I., Shirai S., Kobayashi S., Yabe I., Sasaki H. (2020). A retrospective epidemiological study of tick-borne encephalitis virus in patients with neurological disorders in Hokkaido. Japan. Microorganisms.

[17] Wagatsuma K. (2024). Climate change and expansion of vector-borne diseases in Japan: A public health challenge. New Microbes New Infect.

[18] Miyamoto K. (2002). On an epidemiological study of Lyme disease in Hokkaido. Japan. Med Entomol Zool.

[19] Inokuma H. (2012). The biology of ticks as vectors. J Jpn Soc Clin Bovine Parasitol.

[20] Ohno T., Kato H., Kobayashi Y., Kitaoka M., Kanesaki M., Murai S. (2025). Comprehensive epidemiological analysis of severe fever with thrombocytopenia syndrome in Japan, 2013-2023: descriptive observational study. Lancet Reg Health West Pac.

[21] National Institute of Infectious Diseases (2020). Japanese spotted fever. Infectious Agents Surveillance Report.

[22] National Institute of Infectious Diseases (2022). Tsutsugamushi disease. Infectious Agents Surveillance Report.

[23] Kugeler K.J., Earley A., Mead P.S., Hinckley AF. (2024). Surveillance for Lyme disease after implementation of a revised case definition - United States, 2022. MMWR Morb Mortal Wkly Rep.

[24] Adams J.A., Osasah V., Paphitis K., Danish A., Mather R.G., Russell C.A. (2024). Age- and sex-specific differences in Lyme disease health-related behaviors, Ontario, Canada, 2015-2022. Emerg Infect Dis.

[25] Horiuchi N., Nishigaki Y., Shiwaku K., Matsunaga T., Koike K., Satou E. (2004). Clinical and epidemiological studies of ixodiasis and infectious diseases sequential to *Ixodes* tick bites in rural areas: report I. J Jpn Assoc Rural Med.

[26] Johnson K.O., Nelder M.P., Russell C., Li Y., Badiani T., Sander B. (2018). Clinical manifestations of reported Lyme disease cases in Ontario, Canada: 2005-2014. PLoS One.

[27] Ohira M., Takano A., Yoshi K., Arai A., Aso Y., Furutani R. (2025). Lyme neuroborreliosis in Japan: Borrelia burgdorferi sensu lato as a cause of meningitis of previously undetermined etiology in hospitalized patients outside of the island of Hokkaido, 2010-2021. Eur J Neurol.

[28] Hirsch A.G., Poulsen M.N., Nordberg C., Moon K.A., Rebman A.W., Aucott J.N. (2020). Risk factors and outcomes of treatment delays in Lyme disease: a population-based retrospective cohort study. Front Med.

[29] Rebman A.W., Yang T., Yoon I., Powell D., Geller S.A., Aucott JN. (2023). Initial presentation and time to treatment in early Lyme disease. Am J Trop Med Hyg.

[30] Halsby K., Loew-Baselli A., Strle F., Moniuszko-Malinowska A., Berglund J.S., Cibik V. (2026). Clinical manifestations of lyme borreliosis in Europe: burden of Lyme disease Study (BOLD), 2021-2022. Pathogens.

