# Supplementary figures and images for: Epidemiology of Lyme disease, a growing tick-borne disease of concern, in Japan from May 2013 to March 2024: a descriptive study
Source: IJID Reg. 2026 May 21;19:100919. doi: 10.1016/j.ijregi.2026.100919 (PMC13273715; doi:10.1016/j.ijregi.2026.100919)

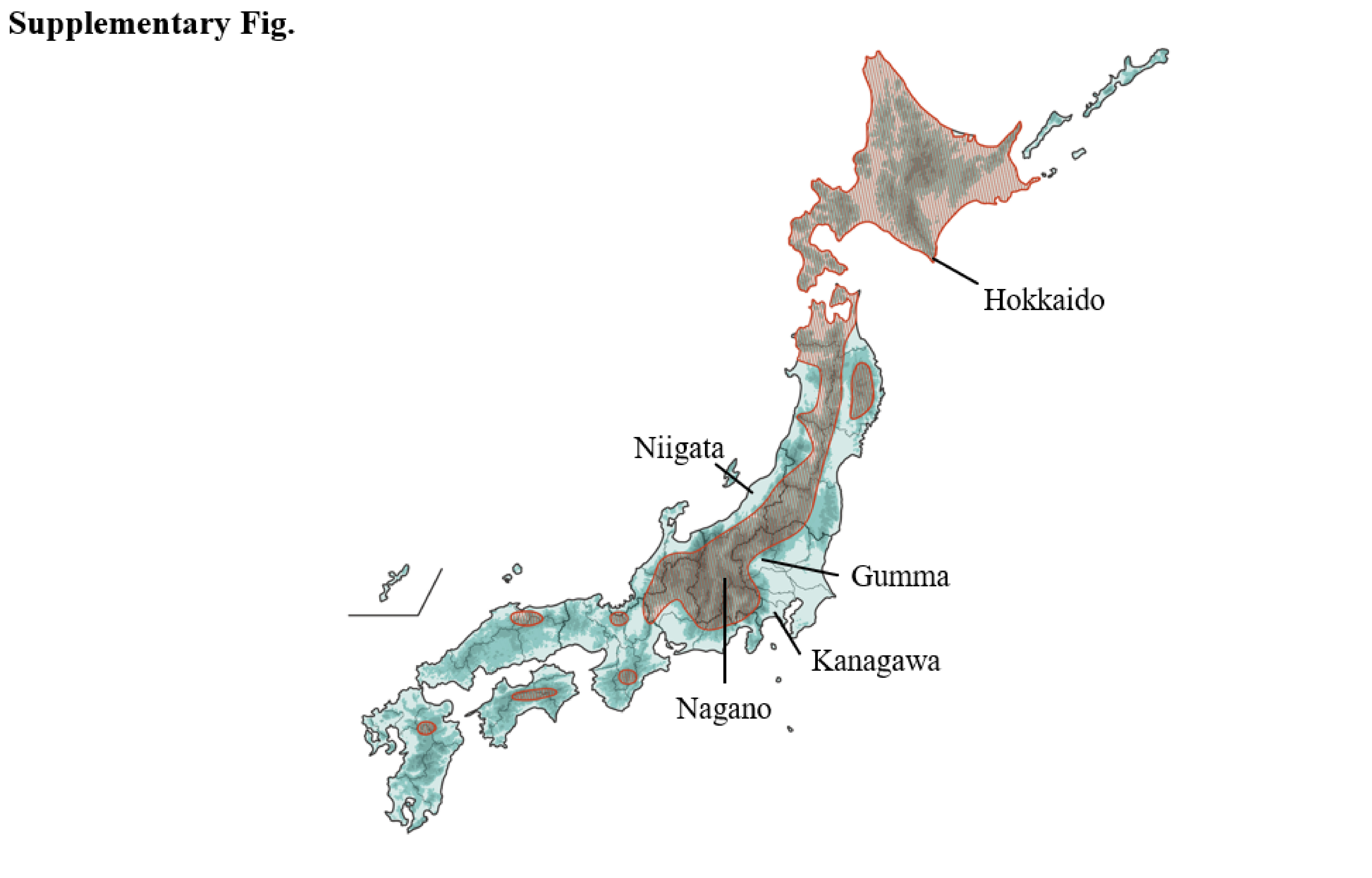

Supplement: Supplementary file 1 — Supplementary Figure 1. Estimated geographic distribution of Ixodes persulcatus in Japan. This figure was created by MEDICAL FIG., a service of Medical Education, Inc., based on the reference (Nobuhiro Takata. Geographic distribution of ticks. Illustrated guide to pathogenic ticks. Kinpodo, INC; 1992. p. 113-135). Red hatched area, estimated habitat of Ixodes persulcatus; shade of green, difference in elevation; thick black line, region boundary; thin black line, prefecture boundary. [file mmc1.jpg]
